# Novel Computational Analysis of Left Atrial Anatomy Improves Prediction of Atrial Fibrillation Recurrence after Ablation

**DOI:** 10.3389/fphys.2017.00068

**Published:** 2017-02-14

**Authors:** Marta Varela, Felipe Bisbal, Ernesto Zacur, Antonio Berruezo, Oleg V. Aslanidi, Lluis Mont, Pablo Lamata

**Affiliations:** ^1^Department of Biomedical Engineering, Division of Imaging Sciences and Biomedical Engineering, King's College LondonLondon, UK; ^2^Arrhythmia Unit—Heart Institute (iCor), Hospital Universitari Germans Trias i PujolBadalona, Spain; ^3^Department of Engineering Science, University of OxfordOxford, UK; ^4^Unitat de Fibrillació Auricular, Hospital Clínic, Universitat de BarcelonaBarcelona, Spain

**Keywords:** atrial fibrillation, computational anatomy, left atrial remodeling, recurrence risk assessment, biomarker classification

## Abstract

The left atrium (LA) can change in size and shape due to atrial fibrillation (AF)-induced remodeling. These alterations can be linked to poorer outcomes of AF ablation. In this study, we propose a novel comprehensive computational analysis of LA anatomy to identify what features of LA shape can optimally predict post-ablation AF recurrence. To this end, we construct smooth 3D geometrical models from the segmentation of the LA blood pool captured in pre-procedural MR images. We first apply this methodology to characterize the LA anatomy of 144 AF patients and build a statistical shape model that includes the most salient variations in shape across this cohort. We then perform a discriminant analysis to optimally distinguish between recurrent and non-recurrent patients. From this analysis, we propose a new shape metric called vertical asymmetry, which measures the imbalance of size along the anterior to posterior direction between the superior and inferior left atrial hemispheres. Vertical asymmetry was found, in combination with LA sphericity, to be the best predictor of post-ablation recurrence at both 12 and 24 months (area under the ROC curve: 0.71 and 0.68, respectively) outperforming other shape markers and any of their combinations. We also found that model-derived shape metrics, such as the anterior-posterior radius, were better predictors than equivalent metrics taken directly from MRI or echocardiography, suggesting that the proposed approach leads to a reduction of the impact of data artifacts and noise. This novel methodology contributes to an improved characterization of LA organ remodeling and the reported findings have the potential to improve patient selection and risk stratification for catheter ablations in AF.

## Introduction

Atrial fibrillation (AF) is the most common sustained cardiac arrhythmia. It is associated with an increased risk of stroke, heart failure, and early death and has a detrimental impact on quality of life, frequently leading to severely disabling symptoms (Calkins et al., 2012). More than 6 million adults in Europe are diagnosed with AF and this number is expected to double by 2050 (Grace and Roden, 2012).

Radiofrequency catheter ablation (RFCA) forms the mainstay of currently available invasive treatments for AF. During RFCA, catheters are introduced into the left atrium, where they are used to cause localized thermal damage to the atrial wall in an attempt to destroy or electrically isolate the areas of abnormal electrical activity responsible for AF. For early AF, RFCA has a medium-term success rate of up to 90%, but in patients with persistent forms of the disease, the success of the procedure drops to less than 70% (Ganesan et al., 2013) and more than half of patients experience additional AF episodes (AF recurrence), requiring multiple procedures to achieve long-term AF termination (Calkins et al., 2012). This reflects an incomplete understanding of the mechanisms underlying AF, leading to a poor identification of the patients most likely to benefit from RFCA procedures.

AF leads to complex alterations (remodeling) at cellular, tissue, and organ level which can contribute to the perpetuation of the condition. Several features of remodeling have therefore been proposed as markers of disease progression to aid the selection of the patients most likely to benefit from catheter ablation. Organ-level alterations can be observed in conventional images using echocardiography, magnetic resonance imaging (MRI) or computed tomography. Previous studies have focused on imaging-based markers of remodeling, measuring atrial dilation using metrics such as the anterior-posterior (AP) radius (Berruezo et al., 2007; den Uijl et al., 2011) and atrial volume (Shin et al., 2008; Abecasis et al., 2009; Hof et al., 2009) or, more recently, left atrial sphericity, a size-independent measure of how closely the LA body resembles a sphere (Bisbal et al., 2013).

It is not clear, however, what LA shape features can best predict AF recurrence before ablation. In two studies, AP radius has been found to independently predict AF recurrence at 12 months post-ablation in echocardiographic or MR images of the LA of over 100 AF patients (Berruezo et al., 2007; den Uijl et al., 2011), above atrial volume, when measured. Other studies, however, reported LA volume to be a better predictor of AF recurrence than AP radius at 1.5 years (Abecasis et al., 2009; Hof et al., 2009) or 6 months (Shin et al., 2008) post-ablation. Finally, in another study, LA sphericity was found to have a superior predictive power to either LA volume or AP radius (Bisbal et al., 2013). Overall, these findings suggest that although LA anatomy can provide important information for pre-RFCA patients' risk stratification, further work needs to be done to determine which LA shape features are most suitable for this purpose.

The personalization of computational cardiac models to clinical data reveals robust and accurate diagnostic and prognostic biomarkers (Lamata et al., 2016). Computational analysis of cardiac anatomy has so far mainly focused in the left ventricle, finding initial evidence of improved risk stratification for sudden cardiac death or pulmonary hypertension (Leary et al., 2012; Vadakkumpadan et al., 2014), but has not yet been consistently applied in the context of AF. The objective of this study is to analyze LA shape in a rigorous consistent framework, in order to exactly determine which LA shape features are more closely associated with the risk of post-ablation recurrence. For this, we encode the LA shape of a cohort of AF patients using smooth (cubic Hermite) meshes. We develop and apply a novel computational framework to identify the LA shape characteristics that can best differentiate between recurrent and non-recurrent patient populations and use it to synthetize extreme recurrent and non-recurrent LA shapes. Using the outcome of this analysis, we propose novel LA shape markers that we exhaustively compare with previously proposed AF recurrence biomarkers, to determine the shape characteristics that can best identify candidates for clinical RFCA therapy.

## Methods

### Subjects and data acquisition

A cohort of 144 atrial fibrillation patients (mean age: 53 years, 24% female), due to undergo an RFCA procedure were imaged under ethical approval using bright-blood cardiac MRI, as detailed in Bisbal et al. (2013). Non-cardiac gated 3D images of the left atrium were acquired at 1.5 T, following the administration of a Gd-based contrast agent, in a single breath-hold, with a reconstructed resolution of 1.4–1.8 mm. The anterior-posterior radius of the LA was additionally obtained from the echocardiographic longitudinal parasternal view in 138 of these patients. Further details concerning image acquisition and post-processing, as well as patient demographics, study enrolment criteria and details of the performed RFCA, can be found in Bisbal et al. (2013). Recurrence was defined to have occurred if there was evidence of an episode of AF or atrial flutter lasting more than 30 s (after a 3-month blanking period). Of the 144 patients, 3 were lost to follow-up before 12 months and an additional 30 before 24 months. 31% of the patients had experienced recurrence in 12 months and 54% at 24 months.

### Creation of smooth left atrial geometrical 3D models

The endocardial surfaces of the left atria of all patients were segmented using the CARTO 3 image integration tool plugin (Biosense Webster, CA, USA) with the pulmonary veins and left atrial appendage excluded at their ostia, as described in Bisbal et al. (2013).

**Figure 1:**
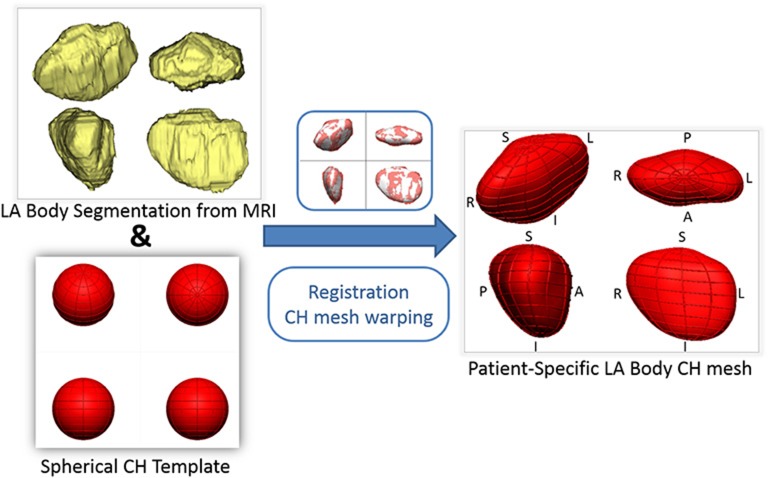
**Outline of the process of generation of patient-specific smooth meshes of the left atrial (LA) body**. The segmentation and meshes are all shown in the same four orthostatic views—F, foot; H, head; A, anterior; P, posterior; L, left; R, right.

To encode the left atrial shape of each subject, a computational 3D geometrical model was fitted to each patient's LA segmentation using a mathematical framework successfully employed to create personalized ventricular meshes (Lamata et al., 2011, 2014). This was achieved in two steps (Figure 1): First, a mesh template of a spherical surface with 144 faces, 134 nodes (vertices) and a radius of 20 mm was created. This mesh template was then registered and warped to the LA segmentation of each patient, following the personalization steps described in Lamata et al. (2011, 2014). The resulting subject-specific meshes are consistent in topology and orientation, so that, for example, node 1 corresponds to the most inferior region of the LA in all subjects.

**Figure 2:**
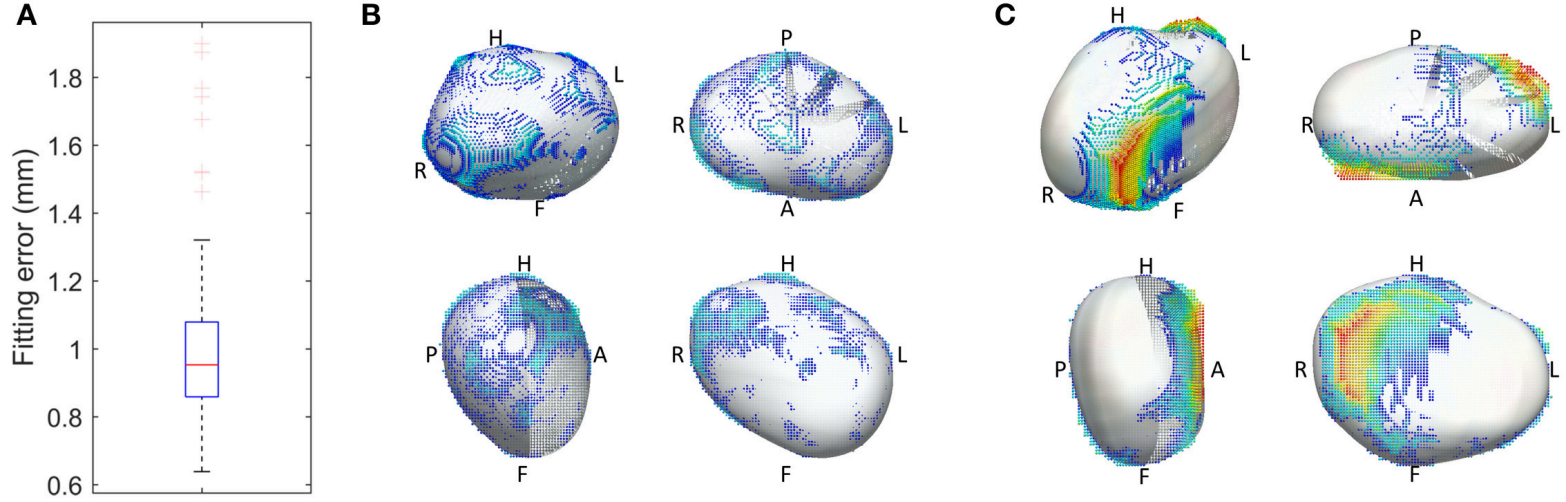
**Geometrical accuracy in the reconstruction of smooth 3D meshes**. **(A)** Fitting error, with the box plot (*n* = 144 cases) of the average L2-norm between the 3D points of the segmentation and the surface of the reconstructed mesh. **(B,C)** Overlay between the 3D mesh (white) and the segmentation, color coded by the distance between them (from blue to red, from 0 to 1 cm respectively), in the best **(B)** and worst **(C)** case of the cohort of 144 meshes.

An important characteristic of the meshes is that they are all smooth due to a cubic interpolation between mesh nodes achieved using class C^1^ cubic Hermite basis functions - the reader is referred to Lamata et al. (2011) for further mathematical details. This is in contrast to linear meshes, where sharp discontinuities at the edge of different mesh elements are permitted. The used meshes are described, in each node, by 12 variables: 3 of these encode the node's position (x, y, and z coordinates) and the other 9 encode partial derivatives of the position to ensure that the atrial mesh is a continuous surface with a smoothly varying surface gradient. This implies that the LA shape of each patient is described by 134 x 12 = 1,608 parameters (degrees of freedom) and that the mesh generation process enforces an implicit shape regularization that eliminates sharp edges, discontinuities and other shape artifacts introduced by the segmentation process. We assessed the accuracy of the mesh fitting by computing the L2-norm between each of the nodes and the segmentation contours (Figure 2).

### Identification of modes of shape variation

From the LA meshes of the 144 subjects, the mean left atrial shape of the cohort was computed using the mean of the 1,608 parameters that encode shape. Mean recurrent and non-recurrent LA shapes at 12 and 24 months post-ablation were similarly computed by averaging the meshes of the patients that had respectively experienced/not experienced recurrence at that time point.

**Figure 3:**
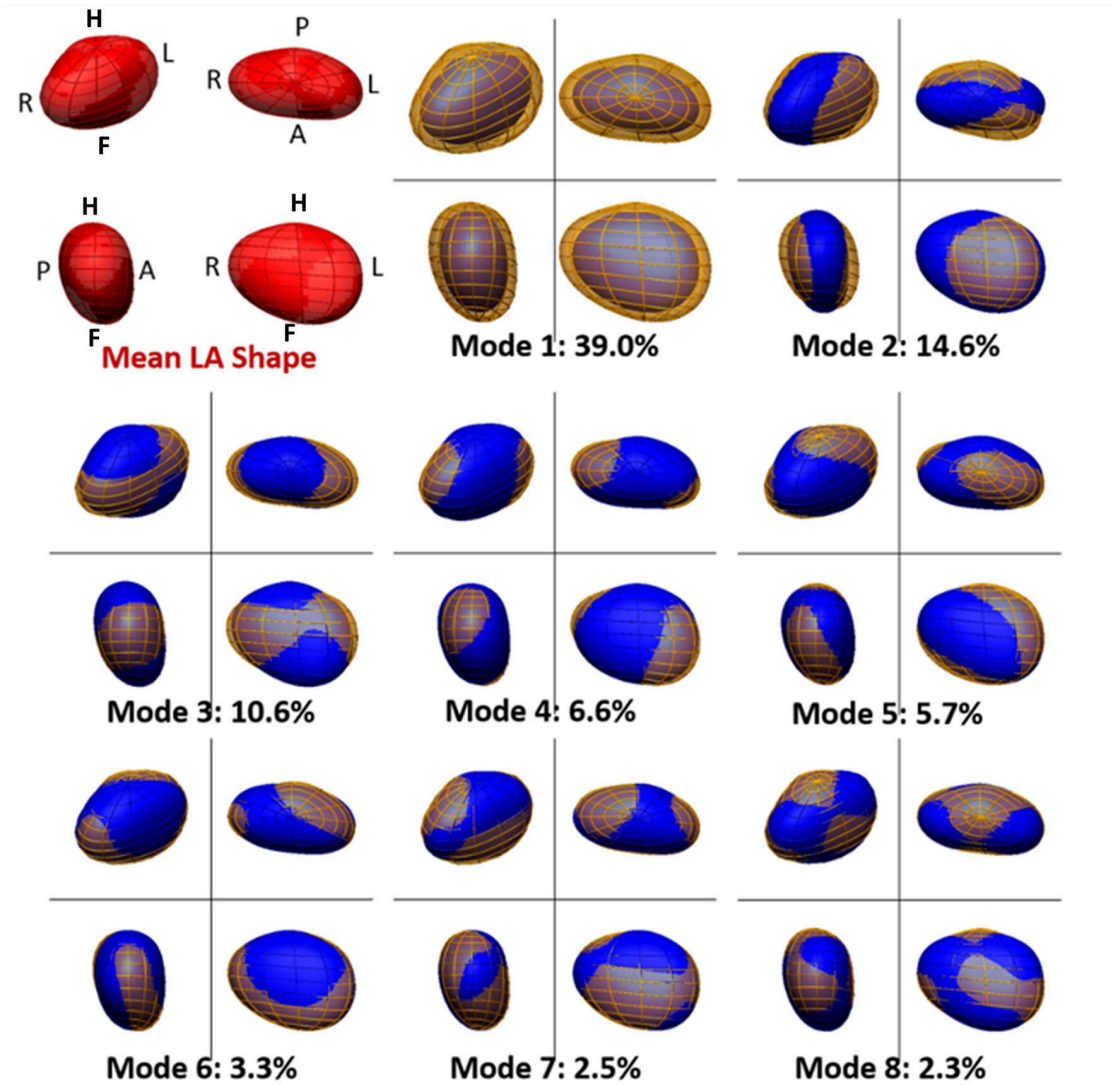
**Mean and main modes of variation of the left atrial shape in the cohort of 144 atrial fibrillation patients**. The mean left atrial shape in the cohort is depicted in red in the top left panel. Other panels depict the LA shape variation encoded by each PCA mode. For each mode, the blue mesh depicts the LA shape obtained by adding, to the mean LA shape, 2 standard deviations (std) of the data along that PCA mode. Similarly, the yellow mesh represents the LA shape obtained by subtracting 2 std of the data along the PCA mode from the mean. The modes are ranked descendingly according to the amount of variation in the data they explain. This is shown, for each mode, as a percentage of total data variation. For each depiction of the left atrium, four views are shown, with the same orientation as in Figure 1.

The analysis and comparison of the 3D LA meshes, each encoded using 1,608 parameters, is unpractical. A technique of dimensionality reduction called principal component analysis (PCA) was adopted to find and rank the directions of anatomical change (called *PCA modes*, see Figure 3) that explain the largest amounts of variance in the data. This enabled us to perform further statistical analyses (such as a linear discriminant analysis, see below) in a low-dimensional space described by the highest rank principal components (instead of a 1,608-dimension space).

**Figure 4:**
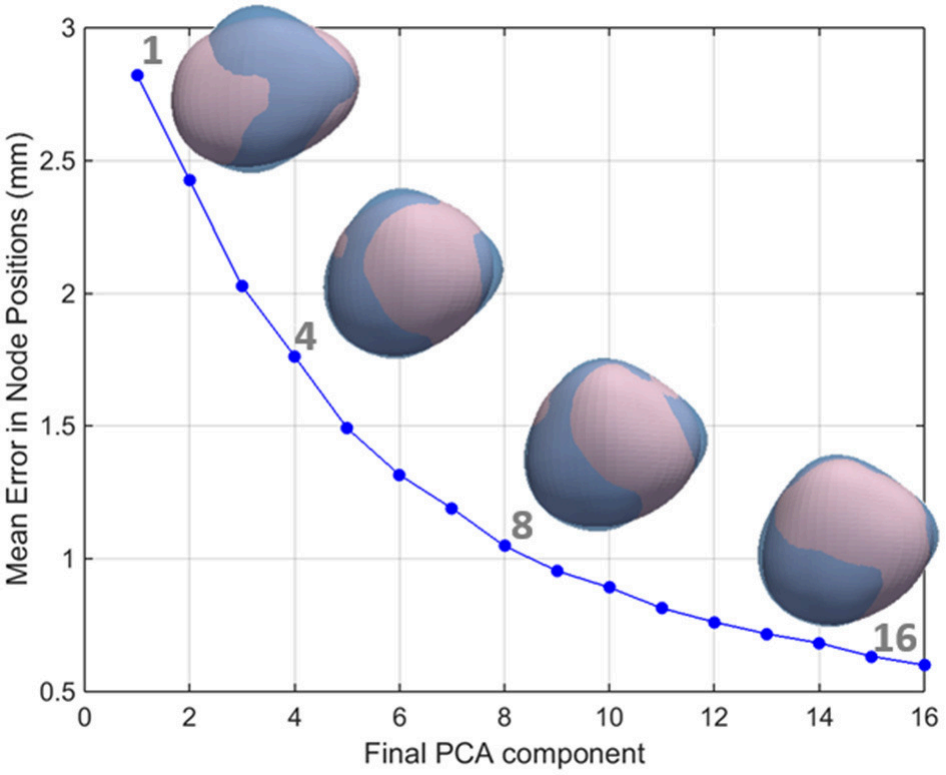
**The relationship between the number of PCA components used and the accuracy in the reconstruction of the left atrial shape**. LA meshes for one representative case are reconstructed in pink using an increasing number of principal components. It can be seen that as the number of components increases, the reconstruction error decreases. As a consequence, the reconstructed pink mesh becomes a better approximation of the fully reconstructed mesh (in blue).

The PCA implementation was based on singular value decomposition performed on centered data and was implemented in Matlab (Mathworks, Natick, MA, USA). PCA modes are mutually orthogonal and, in this case, there are as many PCA modes of variation as there are subjects minus 1 (i.e., 143). PCA modes are ranked in descending order of importance according to the amount of shape variation they explain. In other words, LA shapes can be reconstructed with an increasing level of accuracy starting from the mean shape and sequentially adding the information contained in each subsequent PCA mode (see Figure 4). We note that this study used a cohort of pre-ablation AF patients only and that the captured modes of variation may therefore not be representative of other population segments, such as healthy controls.

We aimed to reject low variance modes that were most likely associated with noise from the image acquisition and segmentation processes rather than true anatomical variability. The number of shape modes chosen to characterize the LA shape was based on the criterion that the mean reconstruction error between the LA mesh and the mesh recreated with the chosen PCA modes should be smaller than 1 mm, corresponding to approximately one half of the MR images' resolution. As such, modes were added to the analysis until an average reconstruction error of 1 mm was reached (corresponding to 8 PCA modes, as shown in the Results section, see Figure 4).

### Optimal differences between recurrent and non-recurrent shapes

To describe the LA anatomical change that best correlates with the risk of recurrence after catheter ablation, we used Fisher linear discriminant analysis (LDA). In our implementation, LDA took the identified subset of the 8 PCA modes and computed the linear combination of these that allowed an optimal separation between recurrent and non-recurrent patients. The combination of PCA and LDA is a common method to regularize the LDA (Belhumeur et al., 1997; Martínez and Kak, 2001), which is ill-posed when the number of data samples (144 in this case) is lower than the number of parameters (here, 1608) used to encode the desired features. In this “high dimension low sample” setting, a poorly-regularized LDA would give a hyperplane for the optimal separation between recurrent and non-recurrent LA shapes that is strongly dependent on noise and therefore non-generalizable. This is illustrated in the Supplementary Material, where LDAs were performed directly on the space encoded by the meshes' degrees of freedom (instead of the PCA space) with two different regularization strategies and were found to give worse-performing predictors than the PCA+LDA approach.

**Figure 5:**
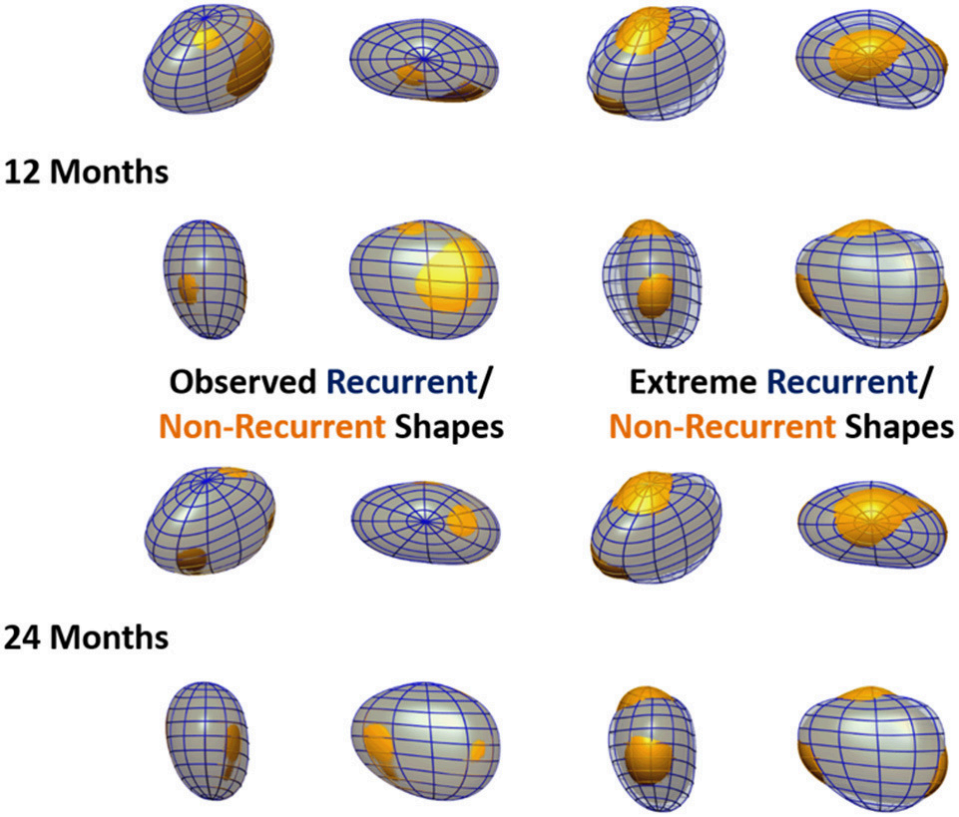
**Observed and extreme recurrent and non-recurrent left atrial shapes**. Recurrent shapes are depicted by a transparent blue wireframe throughout, whereas non-recurrent shapes are shown in yellow. Left: Mean recurrent and non-recurrent shapes, computed using the data in the cohort. Right: Extreme recurrent and non-recurrent shapes, computed by adding/subtracting 2 standard deviations of the data along the oLDA mode to the mean LA shape. Recurrence status is defined at 12 months in the top panels and 24 months in the bottom panels. All meshes are shown in 4 orthographic views, as labeled in Figure 1.

The outcome of the LDA is another anatomical deformation mode, the *LDA mode*, that, instead of maximizing the variance of the population (as the PCA modes), maximizes the differences between recurrent and non-recurrent shapes. As a result of this process, each shape can be characterized by a single score, a coefficient along the direction of anatomical variation described by the LDA, which best classifies recurrence status. The LDA also allows the synthesis of extreme recurrent and non-recurrent LA shapes. This can be done by adding/subtracting the shape change corresponding to 2 standard deviations of the LDA mode to the mean LA shape (see Figure 5).

Two LDA modes were generated in this study. We shall refer to them as the *inclusive* or *optimized* LDA (*iLDA* or *oLDA*) modes respectively, as explained next. The first, iLDA, was motivated by the idea that all PCA modes can potentially have useful anatomical information to predict recurrence. All PCA modes that were considered significant (because they led to a reconstruction error smaller than 1 mm) were included. The second, oLDA, was based on the assumption that there are sources of anatomical variation that do not impact on the prediction of recurrence and, as such, there are PCA modes that degrade, rather than improve, the classification performance in a cross-validation scenario. An exhaustive search of all 247 combinations of the 8 pre-selected PCA modes was performed (i.e., if *n* is the number of significant modes, we then tested all combinations of n taken in groups of size k, with k ranging from 1 to *n*). The best combination in terms of the cross-validation AUC was used to create the oLDA mode.

### Left atrial shape markers

**Figure 6:**
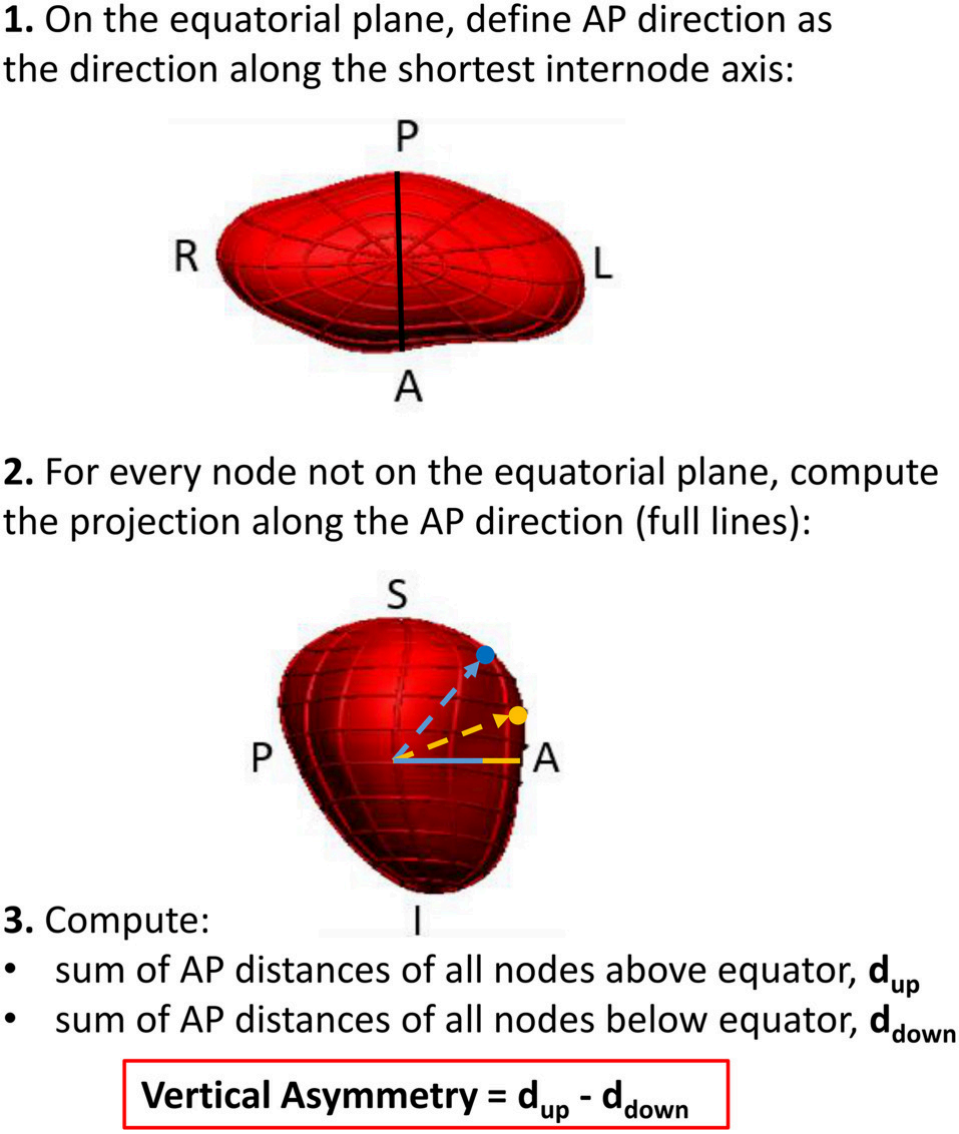
**Computation of the morphological index of vertical asymmetry**.

As it will be reported in the results section, visualization of the extreme shapes encoded by the LDA modes suggested a remodeling outcome associated with an asymmetry of atrial shapes along the foot-head direction (Figure 5). In order to enable an easy interpretation and adoption of this finding in further studies, we analyzed a new marker, vertical asymmetry, computed as described in Figure 6. The absolute value of vertical asymmetry measures the imbalance in AP size between the superior and inferior hemispheres of the LA. A positive vertical asymmetry indicates that the superior hemisphere is larger in the AP direction than in the inferior hemisphere, whereas the opposite holds true for a negative vertical asymmetry.

The search for the atrial shape marker that best predicts AF recurrence included a total of 6 metrics: 3 previously reported metrics computed using the MRI segmentations—LA volume, AP radius and sphericity; and 3 new ones computed from the smooth meshes—iLDA, oLDA (see Optimal differences between recurrent and non-recurrent shapes) and vertical asymmetry. LA volume and sphericity [the normalized sum of the residuals of the best fit of the LA shape to a sphere (Bisbal et al., 2013)] were computed directly from the MRI segmentation.

The search for the optimal metric was comprehensive, testing both each marker individually, and in all possible linear combinations (of 1 to 6 markers, as for the construction of the oLDA described before). The optimal combinations of shape markers were performed using LDA. The objective was to find the optimal single score to predict recurrence that could be computed easily and could thus be used in a clinical setting. The assumption tested is that there can be complementary anatomical information between the different metrics.

The study also included an analysis of the relevance of the imaging modality and pre-processing before the extraction of shape metrics. The commonly used metric of anterior-posterior radius (AP radius) was used for this purpose. This metric was computed in three different manners: direct measurement using echocardiography, measurement from the MRI segmentation (at the level of the center of mass of the LA), and estimation using the shortest equatorial radius from the smooth mesh. As echocardiographic details were missing for 6 patients, this comparison was carried out in 135 and 106 patients at 12 and 24 months post-ablation respectively.

### Statistical analysis

The ability of different shape features to predict post-ablation recurrence was assessed using the receiver-operator characteristic (ROC) curve. The ROC curve characterizes the performance of a metric by graphically showing the true positive detection rate against false positive detection at different cut-offs of the proposed metric. We use the area under the ROC curve (AUC) to allow quantitative comparisons of each shape marker's ability to distinguish between recurrent and non-recurrent LA shapes. In simple terms, the AUC measures the ability of the metric to identify true positives over false positives for a range cut-off values and is therefore less dependent on disease prevalence than other performance classifiers. The higher the AUC of a marker, the better its ability to distinguish between recurrent and non-recurrent cases.

The performance of the classifier was evaluated through a leave-one-out cross-validation test. In this test, each marker is trained on all patient data but one and is then used to predict the recurrence status of the missing case. This is repeated for all cases in the cohort and the AUC of the generated classifier is computed. This cross-validation test provides an indication of how each marker is expected to perform in a cohort different to the one in which it was trained. It can be interpreted as the best indicator of the capability of a marker to predict recurrence for a given subject not included in the cohort in which the marker was trained. This is in contrast to a resubstitution situation, in which the training and testing is done in the same cohort. In the resubstitution situation, cohort-specific, non-generalizable shape features are more likely to play an important role than in the cross-validation scenario.

### Local anatomical changes

Finally, to determine whether significant localized differences in shape exist between recurrent and non-recurrent shapes at different time points, we determined whether there were significant changes in the position of each of the mesh's nodes, by performing Hotelling T-squared tests (at a significance level of 0.01) on node positions after subsequent rigid alignments of all the atrial meshes.

## Results

The personalized LA meshes as well as the patient data for the entire cohort of 144 patients are available in https://dx.doi.org/10.6084/m9.figshare.3851721. The fitting accuracy, measured as the distance between the segmentation contours and the 3D meshes, was 1.00 +/– 0.22 mm (mean +/– std), see Figure 2.

### Main modes of variation of left atrial shape

The mean shape of the LA body is well-approximated by an ellipsoid flattened along the AP direction, with a prominence on the top posterior face, linked to the origin of the left pulmonary veins. Figure 3 shows the average LA anatomy, as well as the LA shapes corresponding to 2 standard deviations from the mean along the 8 main PCA modes. Altogether, these modes explain 84.6% of the variability of the data. The pole-to-pole axis is not strictly foot-head (FH), but deviated leftwards on the inferior face to allow for the insertion of the mitral valve.

Mode 1 explains 39.0% of the variability in the data, as shown in Figure 3, and is strongly linked to LA volume (Pearson's correlation coefficient, ρ_Pearson_ = −0.94). Mode 2 (14.6% of variability) is correlated to changes in the ratio of the left-right (LR) and AP diameters (ρ_Pearson_ = −0.79) and mode 3 (10.6%), with the ratio of the LR and FH diameters (ρ_Pearson_ = 0.87). Subsequent modes are more difficult to be described qualitatively or be mapped to a small number of recognizable anatomical patterns. Note that a positive or negative correlation is not relevant, as a PCA mode is a direction of anatomical change with an arbitrary positive or negative sense.

Individual mesh reconstructions that use all PCA modes up until mode 8 led to an overall mesh reconstruction error (averaged L2-norm of node positions) of just over 1 mm (see Figure 4), corresponding approximately to half of the mean resolution of the original MR images. We therefore used the set of the first 8 PCA modes as the inputs to the LDA.

### Linear discriminant analysis: vertical asymmetry

All first 8 PCA modes were used as inputs to create the iLDA mode. The combination of PCA modes that led to the best discriminative performance in a cross-validation experiment was obtained using PCA modes 1, 2, and 8, which were linearly combined to create the oLDA mode.

The optimal separation direction found by the oLDA defines the extreme recurrent and non-recurrent shapes shown in Figure 5, alongside the observed mean recurrent and non-recurrent shapes as 12 and 24 months post-ablation. (The extreme shapes synthetized using iLDA are qualitatively similar to the oLDA ones and are not displayed for economy of space.) It can be seen that the differences between recurrent and non-recurrent left atria, which are very subtle in the average cases, are greatly magnified when considering the extreme shapes. At 12 months, these changes are dominated by a dilation in the AP direction, as well as a slight shortening left-to-right. These alterations clearly lead to increase in sphericity in the recurrent vs. non-recurrent shapes. Additionally, recurrent shapes show a flattening near the superior pole, alongside a bulging near the inferior pole. These features are also clear at 24 months post-ablation, at which time the alterations in the AP and LR directions associated with sphericity are more modest.

The observed change in the flattening/bulging of the meshes near each vertical pole observed in the extreme shapes suggested the creation of a novel shape marker, vertical asymmetry (see Figure 6), able to distinguish between these two shape features.

### Prediction of AF recurrence

**Figure 7:**
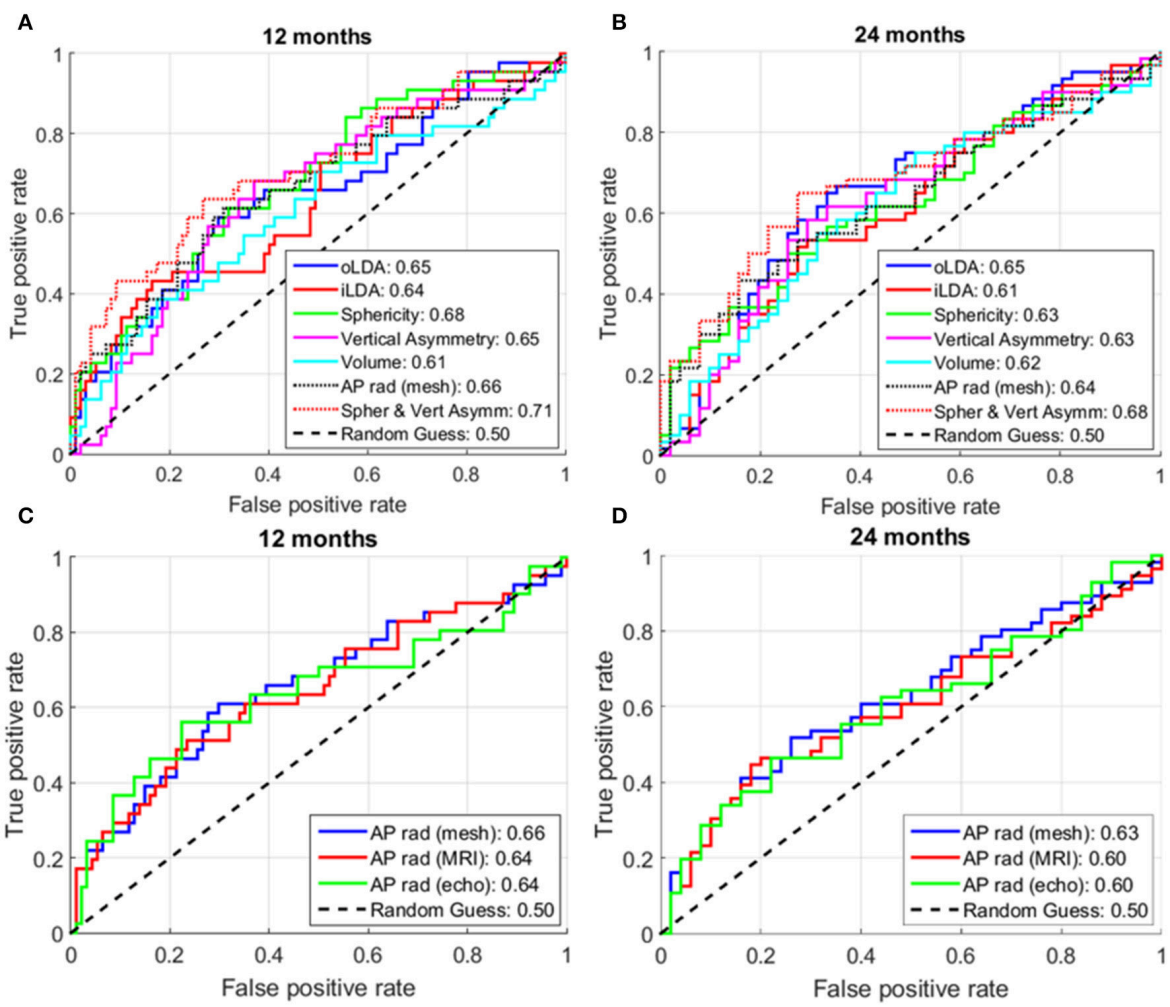
**Receiver operator characteristic (ROC) curves for different analyzed shape markers**. **(A,C)** At 12 months post-ablation. **(B,D)** At 24 months post-ablation. The numbers in the legend show the area under the curve (AUC) for the corresponding shape markers. For each metric, the higher the AUC, the better its ability to distinguish between recurrent and non-recurrent LA shapes. The maximum number of subjects for whom all data was available was used in these analyses, corresponding to: **(A)** 141; **(B)** 111; **(C)** 135; and **(D)** 106 patients.

Figures 7A,B show the ROC curves of the different analyzed shape metrics. It can be observed that, at both 12 and 24 months, sphericity (AUC at 12; 24 months: 0.68; 0.63) and AP radius (0.66; 0.64) are the best single-marker predictors, closely followed by vertical asymmetry (0.65; 0.63). The combination of sphericity and vertical asymmetry shows the highest AUC of all predictors and their combinations (0.71; 0.68), surpassing even the LDA combinations of PCA modes. Of these, the oLDA performs better than the iLDA in a cross-validation situation, with AUCs of 0.65; 0.65, compared to 0.64; 0.61 for the iLDA. The performance of all markers except volume decays at 24 months post-ablation compared to 12 months.

**Figure 8:**
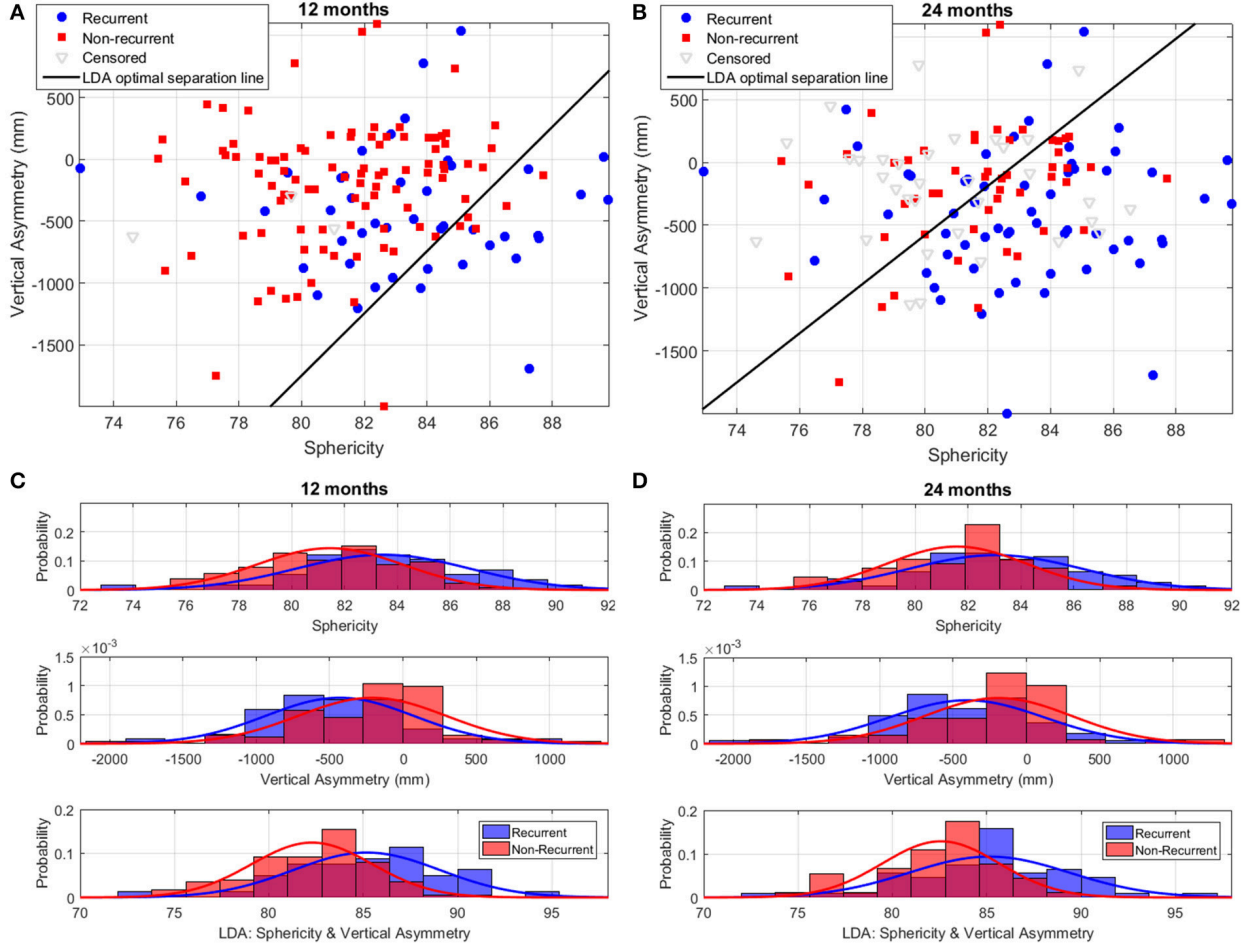
**Discrimination of recurrent and non-recurrent cases using sphericity, vertical asymmetry, and the LDA combination of the two at 12 and 24 months post-ablation**. **(A,B)** Scatter plots of patient data in the sphericity-vertical asymmetry space, showing the optimal separation line between recurrent and non-recurrent cases as computed using LDA. The position of censored (lost follow-up) data is also shown. **(C,D)** Histograms and corresponding Gaussian distribution fits for recurrent and non-recurrent populations according to sphericity (top panel), vertical asymmetry (middle panel), and the LDA combination of the two (bottom panel). The left/right panels depict results at 12/24-months post-RFCA.

Figure 8 illustrates the performance of the LA shape marker found to have the best discriminatory performance in terms of cross-validation AUC: the LDA combination of sphericity and vertical asymmetry. Vertical asymmetry in the cohort was −284 ± 510 mm, with, as expected, a more negative score for recurrent patients compared to non-recurrent ones (Figures 8C,D). The addition of vertical asymmetry to sphericity improves the discrimination between recurrent and non-recurrent patient groups, as can be visually verified by the increased separation between the two Gaussian peaks in the bottom panels of Figures 8C,D compared to the two top ones.

**Figure 9:**
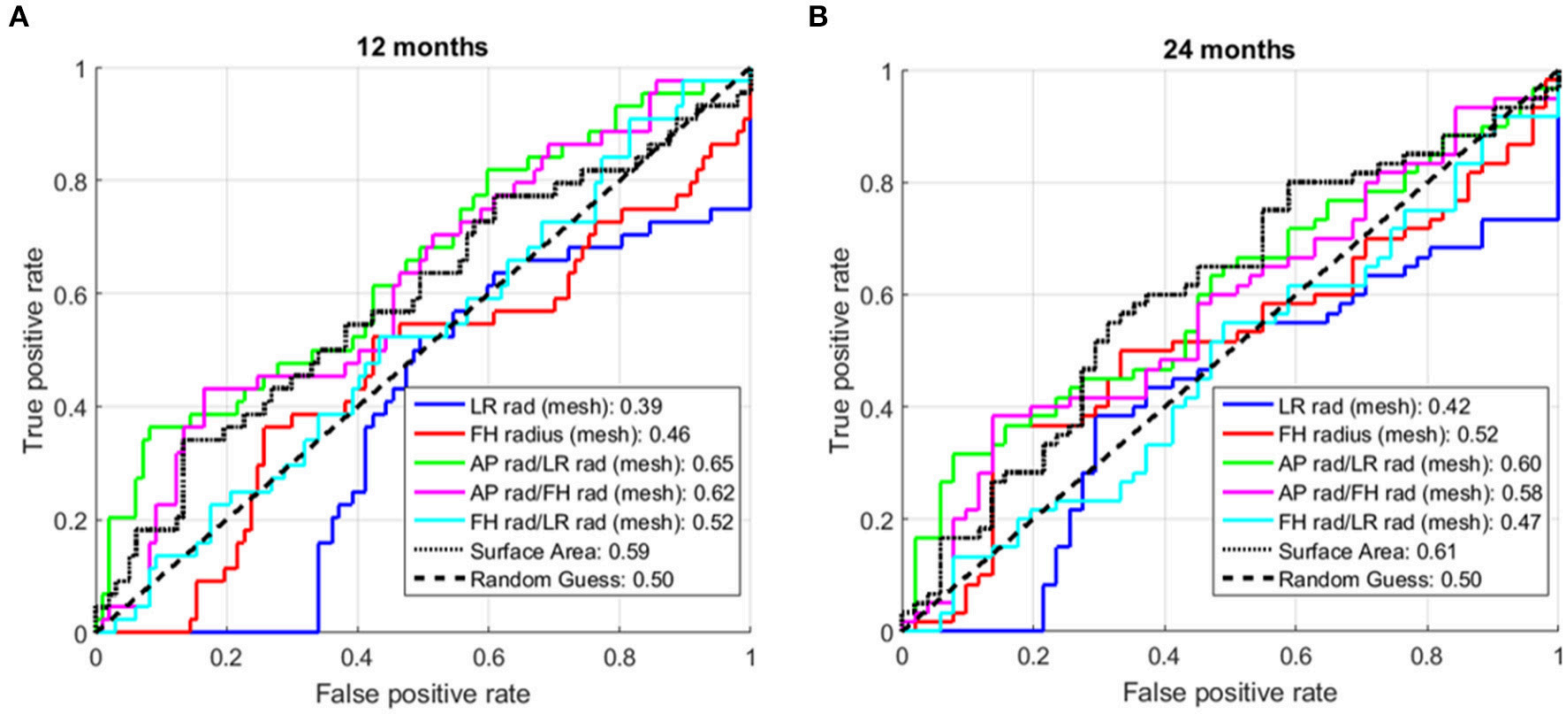
**Receiver operator characteristic (ROC) curves additional shape markers. (A)** At 12 months post-ablation. **(B)** At 24 months post-ablation. The numbers in the legend are the areas under the curve (AUC) for the corresponding shape markers. rad, radius; LR, left-right; FH, foot-head. (All markers showed an AUC>0.5 when applied to the entire cohort. For some markers, the performance drop associated with the cross-validation situation led to a drop in the AUC below 0.5.)

Other simple shape markers, such as foot-head or left-right radius, ratios of radii and surface area were also investigated, but found to have a poor discriminatory performance, as shown in Figure 9.

### Effect of imaging modality and the use of smooth models

We also investigated the predictive power of the AP radius when computed using different modalities or processing techniques. As shown in Figures 7C,D, AP radius estimates computed using the smooth meshes (AUC: 0.66; 0.63) showed a larger predictive power than those computed directly from the segmented MR images or echocardiographic studies (same performance, AUC: 0.64; 0.60 respectively, at both time points).

### Local anatomical changes

Hotelling's T-squared tests failed to find any significant changes in node positions with time, suggesting that shape alterations linked to post-ablation recurrence affect the LA shape in a global rather than localized way.

## Discussion

This work unveils the shape features that can best predict AF recurrence following an RFCA procedure. Using information gained from a statistical analysis of LA shape, we propose a novel marker, vertical asymmetry, which, when combined with sphericity, is found to be the best left atrial shape predictor of recurrence at both 1 and 2 years post-ablation (AUC = 0.71 and 0.68, respectively). The main secondary findings are that recurrence is not found to correlate with any local shape changes and that shape metrics derived from the smooth meshes have a better predictive performance than equivalent MRI or echography-based ones, as shown for the AP radius.

These findings can help in the stratification of AF patients for RFCA procedures and the proposed methodology can be applied to the study of the prognostic value of LA morphology in other atrial pathologies, such as the prediction of embolic cerebrovascular events in mitral stenosis (Nunes et al., 2014). This study demonstrates that smooth computational meshes can extract additional clinical value from the left atrial anatomy captured by medical images. This is a case study of how computational anatomy tools can bring further insights compared to conventional shape measurements.

### LA shape encoded by smooth meshes

This study investigates the structural remodeling of the atria at an organ scale going beyond simple shape metrics (such as volume or AP distance), which are usually estimated from 2D echocardiographic data in clinical practice. Our methodology relies on 3D MR images and smooth meshes, and was designed to more comprehensively analyze atrial shape in AF, comparing these commonly-used clinical shape markers with more complex mesh-based metrics.

The core novelty of the proposed methodology is the use of smooth computational meshes (by cubic Hermite interpolation), making the shape analysis robust to image acquisition and segmentation limitations at the cost of high-resolution anatomical detail. (Note that mesh smoothness can be enforced by other methodologies). The smoothness of the meshes is also useful to capture high-order features of the organ-level shape which are difficult to correctly encode using less smooth (e.g., linear) meshes. An example is the computation of local (Gaussian) curvature, which has been shown to have an important role in the dynamics of the abnormal electrical circuits believed to underlie AF (Rogers, 2002; Dierckx et al., 2013).

It is possible that enforcing smoothness in the mesh fitting process may lead to a loss of valuable anatomical detail present in the images (and therefore in the segmentations). We tested this hypothesis by varying the level of anatomical detail that meshes could capture (imposed by the regularization of the mesh fitting algorithm). Although using a higher level of detail reduced the average segmentation-to-mesh fitting error from 1.00 to 0.59 mm, this did not lead to an increment of the predictive power of the mesh-derived metrics. This suggests that the additional anatomical detail is either not directly relevant to the prediction of AF recurrence (for which large-scale organ-level alterations have traditionally been considered) or that it is contaminated by noise or artifacts.

The choice of smooth meshes to represent LA morphology can prevent localized shape features from significantly contributing to recurrence prediction, as found in this study (section 3.5). Studies that use high-resolution information (such as the encoding of LA shape from cardiac-gate computer tomography images) and meshes that capture higher levels of spatial detail should be better placed to reveal very localized high-resolution remodeling patterns with additional predictive power.

### Statistical analysis of LA shape

PCA and LDA are powerful mathematical tools, but the changes encoded by different modes are not always amenable to simple interpretation. In order to overcome this limitation, most of the variability in the analyzed cohort (Figure 3) was correlated with variations of easily identifiable attributes: Atrial volume for the first PCA mode, followed by changes in the ratios of the different orthogonal radii of the LA for the subsequent two modes.

The synthesis of extreme LA recurrent and non-recurrent shapes (Figure 5) also enables the comparison to previous findings and the generation of novel insights. The extreme shapes show changes consistent with variations in sphericity (particularly at 12 months), as demonstrated in a previous study (Bisbal et al., 2013), and, additionally, alterations in the relative AP size of the superior and inferior LA hemispheres, leading to the definition of a novel shape metric: vertical asymmetry.

### Performance of shape metrics

We hypothesize that LAs with a very negative vertical asymmetry are more susceptible to recurrence, as suggested by the extreme shapes shown in Figure 5. Although, on its own, vertical asymmetry (AUC at 1 year; 2 years: 0.65; 0.63) was found to be slightly inferior to sphericity (0.68; 0.63) when predicting recurrence, a combination of sphericity and vertical asymmetry (0.71; 0.68) was found to be the best predictor of all the analyzed markers, surpassing even the mathematically optimized oLDA metric (0.65; 0.65). This suggests that the insights offered by the visual depiction of the outcomes of the LDA can inform the creation of metrics with a high discriminatory power, either as stand-alone markers or in combination with other shape metrics.

As in any classification problem based on machine learning, we found that increasing the number of PCA modes used as an input to the LDA led to an improvement in discrimination between recurrent and non-recurrent shapes in a resubstitution situation. This was nevertheless a spurious improvement, driven by the fact that training was performed in the same cohort where the algorithm was tested. In a cross-validation situation (where the recurrence status of cases not included in the training dataset is predicted), additional PCA modes do not translate into an improved predictive power. This is exemplified in Figure 7, where the cross-validation AUC (at 1; 2 years) is lower for the iLDA (0.64; 0.61, computed using all first 8 PCA modes) as compared to the oLDA (0.65; 0.65, where only PCA modes 1, 2 and 8 were taken into account).

For similar reasons, a combination of sphericity and vertical asymmetry was found to outperform all other metrics and their combinations, including those where sphericity and vertical asymmetry were combined with additional markers, such as volume or AP radius. We conclude that the addition of more parametric shape dimensions contributes to better explain the differences in a specific population, but cannot be generalized to other cases.

It should be noted that the prediction of AF recurrence is a multi-factorial problem which goes beyond organ-level remodeling. The results of this study, with a peak AUC of 0.71, suggest that remodeling at the organ level should be complemented with other clinical markers to select patients for LA ablation. Clinically, the simplest method is the assessment of an AP radius with echocardiography (AUC of 0.64 and 0.60 at 12 and 24 months). The availability of a 3D shape from MRI and the computation of sphericity improves predictive power (AUC of 0.68 and 0.63) and the suggested combination with a metric computed using the smooth 3D meshes (vertical asymmetry) achieves an additional increment in AUC of similar magnitude (reaching 0.71 and 0.68).

### Effect of imaging and model smoothness on shape metrics

We also analyzed the impact of the imaging modality and the model construction process on the ability of the AP radius to predict post-ablation recurrence. We found that although the AP radius estimated from the smooth meshes had a comparable predictive power to sphericity in all analyzed conditions, its performance worsened when estimates were taken directly from the MRI segmentation or from echocardiographic measurements. This is likely to be linked to a decrease in the precision of AP radius estimates as they are taken from non-smooth MRI segmentations or from operator-dependent echocardiographic measurements. This result highlights the importance of the shape regularization and consistency in orientation enforced by the personalized smooth computation meshes in the creation of robust shape metrics. These findings do not apply to other metrics such as volume, which are less sensitive to the regularization imposed by the smooth mesh creation.

### Comparison with previous studies

Our findings can be related to another computational analysis study found in the literature. Previous work by Cates et al. (2014) encoded left atrial shape by creating a point-based model of the endocardial LA wall, which was manually segmented from bright-blood MR images of 137 subjects. The pulmonary veins were removed, but not the LA appendage. Significant differences in LA shape between control, paroxysmal and persistent AF patients were found. Furthermore, the authors determined the main modes of shape variation using PCA, but restricted their analysis to a single PCA mode, which was found to be significantly different among the three subject groups and linked to a normalized AP radius. This previous study therefore corroborates the finding that alterations in the AP dimension are linked to LA organ-level remodeling in AF. It does not, however, establish any links between LA shape and post-ablation recurrence and does not compare the ability to characterize remodeling in AF of this PCA mode to that of conventional shape markers such as AP radius.

### Mechanisms of recurrence

Our study demonstrates statistical correlations between the LA shape metrics and AF recurrence in a large cohort of patients. The implicit hypothesis is that the morphological remodeling at the organ level is a sign of disease progression and a metric for risk stratification.

However, the possible biophysical pathways linking atrial shape and AF recurrence have not yet been elucidated. Possible mechanisms can include the initiation of AF via stretch-activated channels due to atrial dilation, its perpetuation mediated by the slow movement of abnormal electrical circuits in regions of high atrial curvature (Dierckx et al., 2013) or biomechanical adaptions due to a loss in atrial contractility. Such mechanisms are not straightforward to explore in a clinical setting. Future studies will aim to elucidate these by exploring the impact of LA shape on atrial arrhythmogenesis using computational biophysical models of the atria (Aslanidi et al., 2011).

### Limitations

As in the original study in which this cohort of patients was analyzed (Bisbal et al., 2013), different ablation sets were applied based on the physician's criteria. Pulmonary vein isolation was performed in all patients and further substrate modification (such as the LA roof or mitral isthmus lines) were targeted in some cases.

Furthermore, the performed analysis only considered the shape of the left atrial body. This leaves out the anatomy of the left atrial appendage and pulmonary vein insertions and also of the right atrium, which could also carry important information concerning recurrence risk. However, the proposed methodological approach, which prioritizes robustness through the choice of a smooth LA representation, is not well-suited to capture fine anatomical details and to account for the large inter-individual anatomical and topological variability that these structures can present.

The performed MRI acquisition was not cardiac gated, implying that the analyzed shape carries contributions from several phases of the cardiac cycle. Given that the weighting given to each phase of the cardiac cycle varies from patient to patient, this may introduce an important source of variability into the analyzed LA shape. Future work will focus on applying the developed framework to cardiac-gated MRI to help clarify which phases of the cardiac cycle provide the most useful information for AF patient stratification in the RFCA context.

This work presents one of the possible approaches to balancing the extraction of useful information and the degradation by noise and artifacts in a given image segmentation. As reported in the Supplementary Material, the proposed methodology reached the best performance and clearest interpretation of results in our cohort of 144 cases, but there are methodological decisions that may be improved by more robust criteria in future studies. These include the number of the PCA components that should be used to describe shape accurately, the regularization strategy for an LDA and the highest spatial content that the personalized meshes should capture (i.e., the level of anatomical detail).

Finally, both the presence and pattern of LA fibrosis and atrial wall thickness have been shown to be an important factor in both arrhythmia mechanisms and patient stratification for sudden cardiac death and hence including information about fibrosis or atrial wall thickness from patient-specific MRIs (Marrouche et al., 2014; Varela et al., 2015) is likely to enhance future computational analyses of atrial shape in the context of AF.

In conclusion, we present a methodology to rigorously encode LA shape and use it to investigate shape predictors of AF recurrence following RFCA. LA vertical asymmetry, a novel shape marker, led, in combination with sphericity, to the best predictive power for recurrence at 1 and 2 years. This methodology has the potential to improve AF patient selection for RFCA and to lead to an improved understanding of the organ-level remodeling process in these patients.

## Author contributions

MV wrote the data analysis software, analyzed, and critically reviewed the data and drafted the manuscript. AB, FB acquired the data. OA, FB, LM contributed to the study design. EZ contributed to the data analysis. PL designed the study, wrote the software that creates the patient-specific meshes and atlas and did part of the data analysis. All authors critically reviewed the manuscript and gave final approval for publication.

## Funding

This work was supported by: The UK Department of Health (via the NIHR comprehensive Biomedical Research Centre award to Guys & St. Thomas NHS Foundation Trust in partnership with KCL and King's College Hospital NHS Foundation Trust and the Healthcare Technology Co-operative for Cardiovascular Disease); the Wellcome Trust-EPSRC Centre of Excellence in Medical Engineering [WT 088641/Z/09/Z]; the British Heart Foundation [PG/15/8/31130] to MV and OA; the Wellcome Trust and the Royal Society [WT 099973/Z/12/Z] to PL; the H2020 EU Framework Programme for Research and Innovation [655020-DTI4micro-MSCA-IF-EF-ST] to EZ and a grant by La MARATO - TV3 (ID 201527) to FB.

### Conflict of interest statement

The authors declare that the research was conducted in the absence of any commercial or financial relationships that could be construed as a potential conflict of interest.

## Abbreviations

AF: Atrial Fibrillation
AP: Anterior-Posterior
AUC: Area Under the Curve
FH: Foot-Head
iLDA: inclusive LDA
LA: Left Atrium
LDA: Linear Discriminant Analysis
LR: Left-Right
MRI: Magnetic Resonance Imaging
oLDA: optimized LDA
PCA: Principal Component Analysis
RFCA: Radio Frequency Catheter Ablation
ROC: Receiver Operator Characteristic.
