# Supplementary material for: Novel Computational Analysis of Left Atrial Anatomy Improves Prediction of Atrial Fibrillation Recurrence after Ablation
Source: Front Physiol. 2017 Feb 14;8:68. doi: 10.3389/fphys.2017.00068 (PMC5306209; doi:10.3389/fphys.2017.00068)
Supplement: Supplementary file 1 [file DataSheet1.pdf]

Supplementary Material

Novel computational analysis of left atrial anatomy improves prediction of atrial fibrillation recurrence after ablation

Marta Varela, Felipe Bisbal, Ernesto Zacur, Antonio Berruezo, Oleg V. Aslanidi, Lluis Mont, Pablo Lamata*

*** Correspondence:** Dr Pablo Lamata: pablo.lamata@kcl.ac.uk

1 Supplementary Data

1.1 Choice of LDA Regularization

A Principal Component Analysis (PCA) previous to a Linear Discriminant Analysis (LDA) is a useful approach commonly used in the literature for regularization of the LDA. If f is the number of features used in the classification task (1608 degrees of freedom in this case), the LDA requires at least $f+1$ training cases (1609 compared to 144 in this study) to guarantee that the matrix is not singular. To solve this issue, the LDA needs to be regularized.

In our problem the degrees of freedom of the computational mesh are heavily correlated, and this annex investigates the impact of different choices of the LDA regularization. The use of an intermediate shape space, the PCA space, as detailed in the main manuscript, is one possible solution.

Methods: An LDA of the mesh degrees of freedom is perform, without a preliminary PCA. Three possibilities are evaluated:

(1) LDA without any regularization,

(2) the inversion of the covariance matrix using the pseudo-inverse,

(3) the use of the diagonal of the covariance matrix.

Results and Discussion: Depending on the choice of regularization we have been able to identify qualitatively two possible solutions that split the population of 144 meshes, illustrated in Supplementary Figure 1:

- **Methods (1) or (2) (overfitted result):** the LDA achieves a perfect, or almost perfect split of the population in resubstitution (AUC ~ 1), but a very poor performance in cross-validation (AUC ~ 0.5). The illustration of the extreme shapes encoded by the LDA mode displays very small changes in the anatomy (see top row in Figure A.1). This is an example of the "high dimension low sample" problem discussed in the main manuscript.

- **Method (3) (reasonable result):** using the diagonal of the covariance matrix the LDA obtains a positive classification outcome in cross-validation (leave-1 out, 0.62 and 0.58 at 12 and 24 months respectively), but below the performance of the PCA+LDA (0.65 and 0.65 of the oLDA, see main manuscript). Qualitatively the extreme shapes of the LDA mode at both time points, 12 and 24 months, are quite similar between them, but do not clearly show the changes in AP distance, sphericity or vertical asymmetry observed when using the PCA+LDA approach. We interpret that the loss of discriminative power and detail is driven by the loss of information through the process of only retaining the elements of the diagonal of the covariance matrix.

Conclusion: The LDA regularization choice has an impact on results. The PCA + LDA strategy provides the best performance in terms of predictive power and interpretation of the remodeling pattern.

1.2 Choice of PCA Modes

In this part, we investigate how the predictive power of LDA depends on the truncation of the PCA performed prior to the LDA.

Methods: An exhaustive search of the optimal performance of the LDA over the PCA modes with different choices of the PCA modes to include was performed. The choice of PCA modes to include varied between choosing the 3 first modes to all first 15. Performance is evaluated by a leave-1 out AUC at both 12 and 24 months.

Results and Discussion: No choice of PCA modes improved the cross-validation AUC compared to the choice of modes 1, 2 and 8 reported in the main manuscript. This result suggests that there is no further useful information for the purposes of recurrence prediction contained in the smaller modes beyond mode 8.

For illustrative purposes, we show the outcome of the LDA performed on the combination of modes 1, 2 and 5. This reveals a remodeling pattern where the extreme recurrent shape has very large: volume, AP distance, and sphericity, as reported in the literature (see Figure A.2 panel A).

The remodeling pattern captured with this choice of PCA modes (1, 2, 5) shows a larger deviation in shape relative to the mean than the oLDA (optimal combination of modes 1, 2 and 8) reported in the manuscript. This is because PCA mode 8 encodes a lower shape variance than mode 5. As modes with smaller variance gain a higher weight in the LDA, the extreme shapes computed deviate less from the mean shape.

Conclusion: The choice of PCA modes 1, 2 and 8 provided the best discriminative performance and allowed for the observation of remodeling patterns beyond those explained by volume, AP distance and sphericity, namely by the metric we dubbed vertical asymmetry.

2. Supplementary Figures

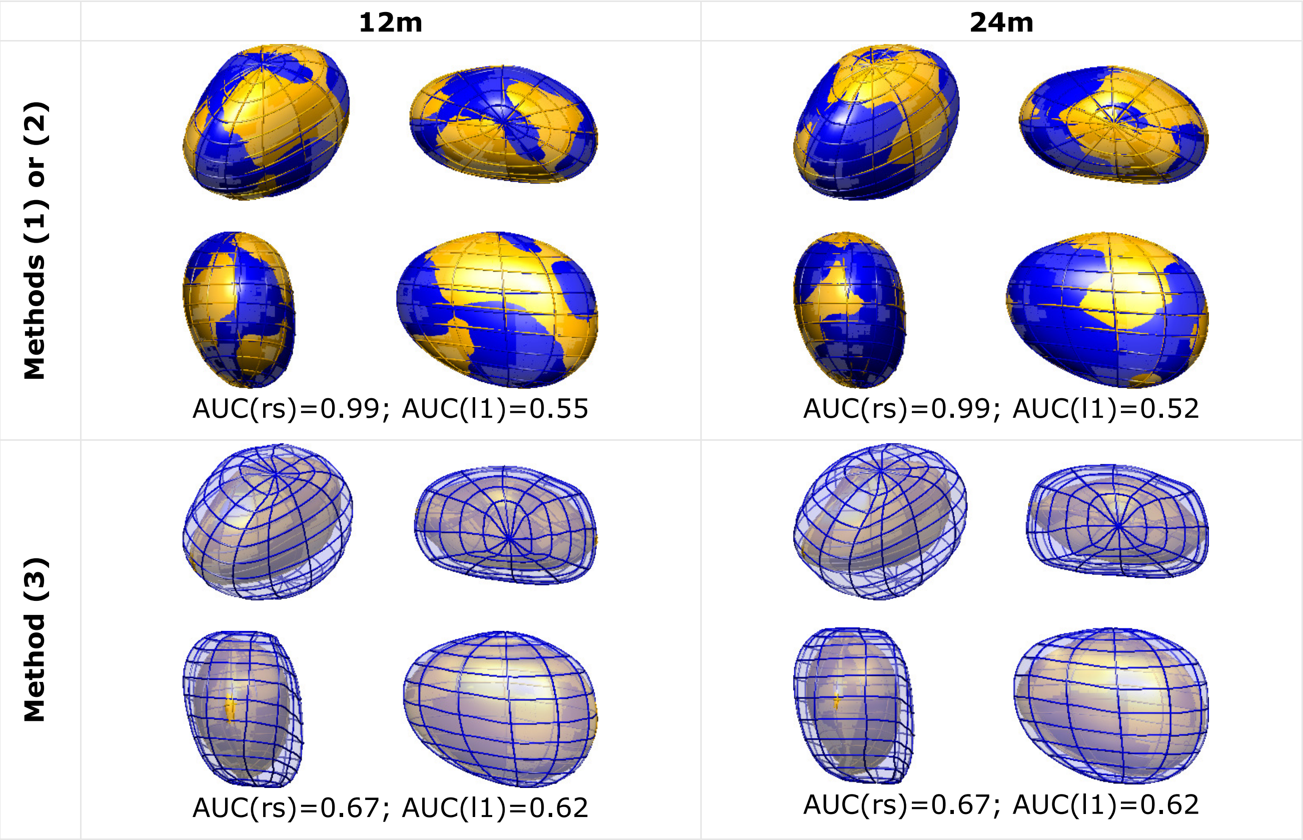

Supplementary Figure 1. Results of the LDA on the original mesh degrees of freedom. Each cell shows the overlay of the two extreme shapes described by the LDA mode at +/- 3 standard deviations, the blue one corresponding to the extreme recurrent, and the yellow to the extreme non-recurrent (top row with both meshes opaque colours, and bottom row with the blue mesh semi-transparent for ease of visualization). Metrics reported in each panel are values of the Area Under the Curve (AUC) of the Receiver-Operator Curve: first for the resubstitution test (rs), second for the leave-1-out cross validation test (l1).

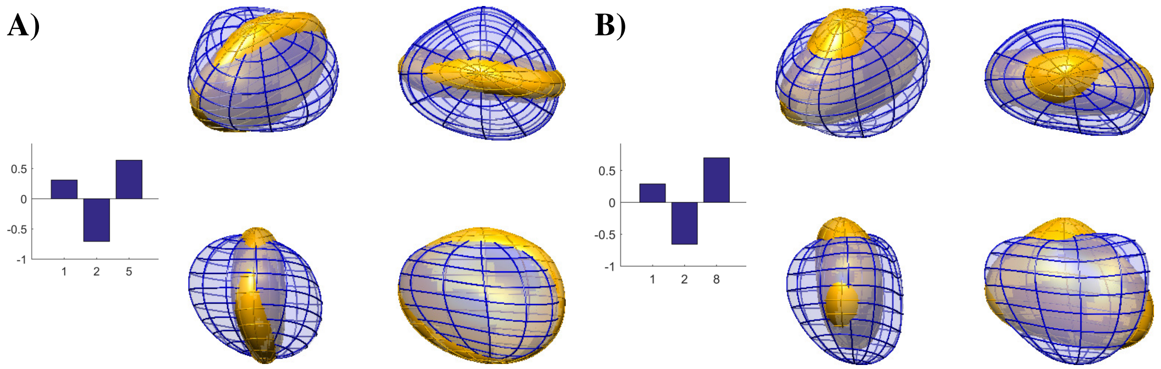

Supplementary Figure 2. LDA analysis including modes 1, 2 and 5 (panel A) and modes 1, 2 and 8 (panel B) at 12 months. Each panel shows the linear coefficients found for each mode and the extreme shapes synthetized at +/- 3 std, with the blue semi-transparent mesh corresponding to the extreme recurrent, and the yellow opaque to the extreme non-recurrent.
